# Effects of game design characteristics of a virtual reality serious game for upper-limb prosthesis control training on motor learning

**DOI:** 10.3389/fresc.2025.1520184

**Published:** 2025-06-17

**Authors:** Jack Tchimino, Bart Maas, Bram van Dijk, Alessio Murgia, Corry K. van der Sluis, Raoul M. Bongers

**Affiliations:** ^1^ Department of Human Movement Science, University of Groningen, University Medical Center Groningen, Groningen, Netherlands; ^2^ Department of Rehabilitation Medicine, University of Groningen, University Medical Center Groningen, Groningen, Netherlands

**Keywords:** prosthesis training, prosthesis control, EMG control, virtual reality, serious games, motor rehabilitation

## Abstract

**Introduction:**

Rehabilitation outcomes of hand prosthesis control training generally benefit from an early start in the rehabilitation regimen as well as the use of modern technologies, like serious games, in lieu of more conventional methods. In this study, we tested a virtual reality based myoelectric prosthesis training serious game, specifically how the game design elements affected different aspects of motor control and training.

**Methods:**

Ten able-bodied participants were asked to execute a series of tasks by controlling an LDA-controlled 1-DoF virtual hand prosthesis within a virtual reality environment (VRE) serious game. The tasks included grasping and manipulating virtual objects and controlled force modulation, the latter facilitated by artificial feedback communicated to participants in the VRE.

**Results:**

The results indicated that the game physics affected the way that the participants completed the tasks, while the tasks themselves appeared to be approached and executed in different ways by the participants. Contrary to expectations, however, the force feedback did not influence the participants' ability to precisely modulate the grasping force applied with the virtual prosthesis.

**Discussion:**

Based on these results, it can be concluded that users can be trained within the proposed framework to develop motor skills that might be translated into the use of a real prosthesis, in a more engaging and timely manner.

## Introduction

Loss of an upper limb is a traumatic, life-altering event, with far-reaching consequences on the quality of life of those affected (1). Myoelectric prostheses offer functional and cosmetic restoration of a missing limb. Learning to control such a device requires a prolonged rehabilitation and training procedure which, however, is missing sufficient standardization and whose results lack scientific validation (2). Once the user has been fitted with a prosthesis, the training most usually involves them repeatedly visiting a certified therapist, who guides them through various tasks of a control training regimen.

It has, nevertheless, been reported that the tasks performed during these sessions can be repetitive, unengaging, and characterized by low motivation on the side of the users (3). More importantly, despite being designed to teach the users how to operate their prosthesis, the training tasks do not always correspond to activities of daily living, which might affect the effectiveness of the training with respect to prosthesis use post-rehabilitation (4). Additionally, as evidence suggests the decrease of neuroplasticity over time post-injury (5, 6), initiating the rehabilitation protocol earlier, during the pre-prosthetic phase, may yield better outcomes. However, in the case of amputations, the wound must be allowed time to heal and residual limb edema must be reduced before the user can be fitted with a prosthesis and the rehabilitation program can begin.

Serious games are digital applications designed to achieve an additional goal besides purely to entertain (7), by motivating the player to meet said goal through a fun and engaging experience. They have, thus, been identified as potential candidates for inclusion in healthcare training applications, both for providers and patients (8). More specifically, a field for which serious gaming has been extensively explored is that of motor learning, for example for stroke rehabilitation (9–11) and myoelectric prosthesis control training (4, 12–15).

More recently, serious game interfaces have included the use of virtual reality, with the users performing the game tasks within a virtual environment. A virtual reality application includes the introduction of users into an artificial environment, displayed to them through a suitable headset. The user can move freely in the virtual environment and interact with it using handheld controllers. Despite being marketed mainly as an entertainment tool, VR has been explored for use in a wide range of motor control learning applications within the healthcare field, including prosthesis control training (16–18). In this field of application, it has been argued that the design opportunities offered by the virtual environment can address several of the issues that conventional rehabilitation protocols face, as listed above.

For instance, the enhanced engagement of a user with a serious game (usually displayed on a conventional computer monitor) can be further amplified by the immersive nature of the virtual reality game, while at the same time the game tasks can be designed to closely mimic real-world scenarios. In this way users may find their training to be more relevant to activities of daily life than conventional prosthesis training (exploiting simple repetitive relocation tasks). Finally, a properly designed system may eliminate the requirement of the fitting and manufacturing of a complete prosthesis for training, allowing the user to initiate their training early in their rehabilitation journey, taking advantage of the increased neural plasticity that characterizes the period immediately following the injury. VR serious games have been shown to be more engaging in task training (19, 20). Despite the promise that such systems have shown in the scientific literature concerning their application in prosthesis rehabilitation, there appears to be no consensus regarding their standardization in terms of design and focus (21). Such specifications can be defined by considering the desired rehabilitation outcomes and training goals, as well as the user experience. This insight can inform the design and development of VR serious games aimed at prosthesis control training.

To that end, this study uses a VR prosthesis control training game to evaluate the performance of participants over a multi-day training regimen. In the game, the player assumes the role of a barista at a seaside café, where they must use a virtual prosthesis to prepare different types of beverages, with the size and physical characteristics of each beverage type implementing a different game task. The prosthesis in the VR environment is controlled through a myoelectric interface with EMG signals recorded from the participants' forearms, identical to the way a real prosthesis is controlled, as will be described in detail in the Materials and Methods section.

The goal of this study was to observe the performance of the participants over the training sessions and identify ways in which the constituent elements of this system can be more effectively exploited to promote optimal prosthesis control as well as a more engaging training scheme. Several aspects of the game are implicitly evaluated: namely, the physics of the virtual environment (i.e., the physics engine of the game, which does not fully align with real-world physics), the quality of the myoelectric control scheme, the game tasks, and the provision of feedback, following the categorization presented in (21). It is of great importance to note that our companion study (22) has demonstrated the efficacy of this training system, in terms of motor learning and skill transfer as well as user motivation, while Rozevink et al. (23) evaluated the system in terms of usability and motivation. These outcomes are, therefore, beyond the scope of this pilot usability study, which, rather, explores the serious game design methods. Specifically, by monitoring user performance during gameplay, this study evaluates the way that different game characteristics affect the training, with no claims to its efficacy, beyond those made in the two companion studies.

To be able to make informed and specific recommendations on the design and focus of the training system, the following hypotheses were put forward regarding several outcome measures, each relating to one of the four aspects distinguished before. The participants were expected to react and adapt to the altered physics of the virtual world, despite the sensory mismatch between the virtual world and their own proprioception. Moreover, from a myoelectric control standpoint, the training was expected to influence the duration of the grasping action with the virtual prosthesis, with the participants performing the task faster by the end of the training. The participants were expected to improve their force modulation skills, an outcome quantified by the rate at which they crushed fragile virtual objects during the training. Additionally, they were expected to execute prosthesis movements in a more fluid and controlled manner, as opposed to a stepwise motion, by the end of the training.

These outcomes can provide insight into the effect of this system on the participants' motor skills development, determining its effectiveness on prosthesis control training. In turn, this insight can be used to identify ways in which a virtual reality serious game can be improved for optimal rehabilitation results.

## Materials and methods

### Participants

For a person to participate in this experiment, they had to be above 18 years of age, right-handed, with normal or corrected eyesight, and without upper-limb injuries (able-bodied). 10 participants were recruited from the student population of the UMCG to perform the experiment, 4 male and 6 female of 20.6 ± 1.4 years. The participants had read the detailed information letter prior to joining the experiment, which was also explained in detail by the experimenters. The participants signed an informed consent form before commencing the experiment. This study was performed in accordance with Ethical Protocol RR11470.

The right-handedness criterion was evaluated by having potential participants fill in the Edinburgh Handedness Inventory (24). A score of below 50% right-handedness indicated that the participant would not take further part in the experiment. The mean score of the participants was 88 ± 9.8 right handedness.

The participants also completed the Motion Sickness Susceptibility Questionnaire (MSSQ) (25), which evaluated their proclivity towards motion sickness. This was done to ensure that the participants would not experience cybersickness during the experiment, due to visuo-vestibular sensory information mismatch (26). We regarded a high score in the MSSQ as an indication that a participant would likely experience cybersickness while in the virtual environment, which would eliminate them from the pool of participants for safety reasons. In the interest of practicality, we only considered the adult scores of the MSSQ and used half of the population median [11.3 (25)], as a cutoff score to allow the participants to perform the experiment.

### Experimental setup

The experimental setup consisted of the following components: (1) A Meta Quest 2 virtual reality headset (Meta Platforms Technologies, CA, USA), (2) 8 pairs of dry dome electrodes (domes: 13Z161-3, electrodes: 13E400, OttoBock, Duderstadt, DE), embedded in an elastic cuff (Myo Plus diagnostic cuff, 757M20, OttoBock, Duderstadt, DE) and connected to a controller (13E520, OttoBock, Duderstadt, DE), (3) a wrist immobilization orthopedic splint, a tennis ball, and medical tape (4) a Samsung A8 tablet, and (5) a standard laptop.

**Figure 1:**
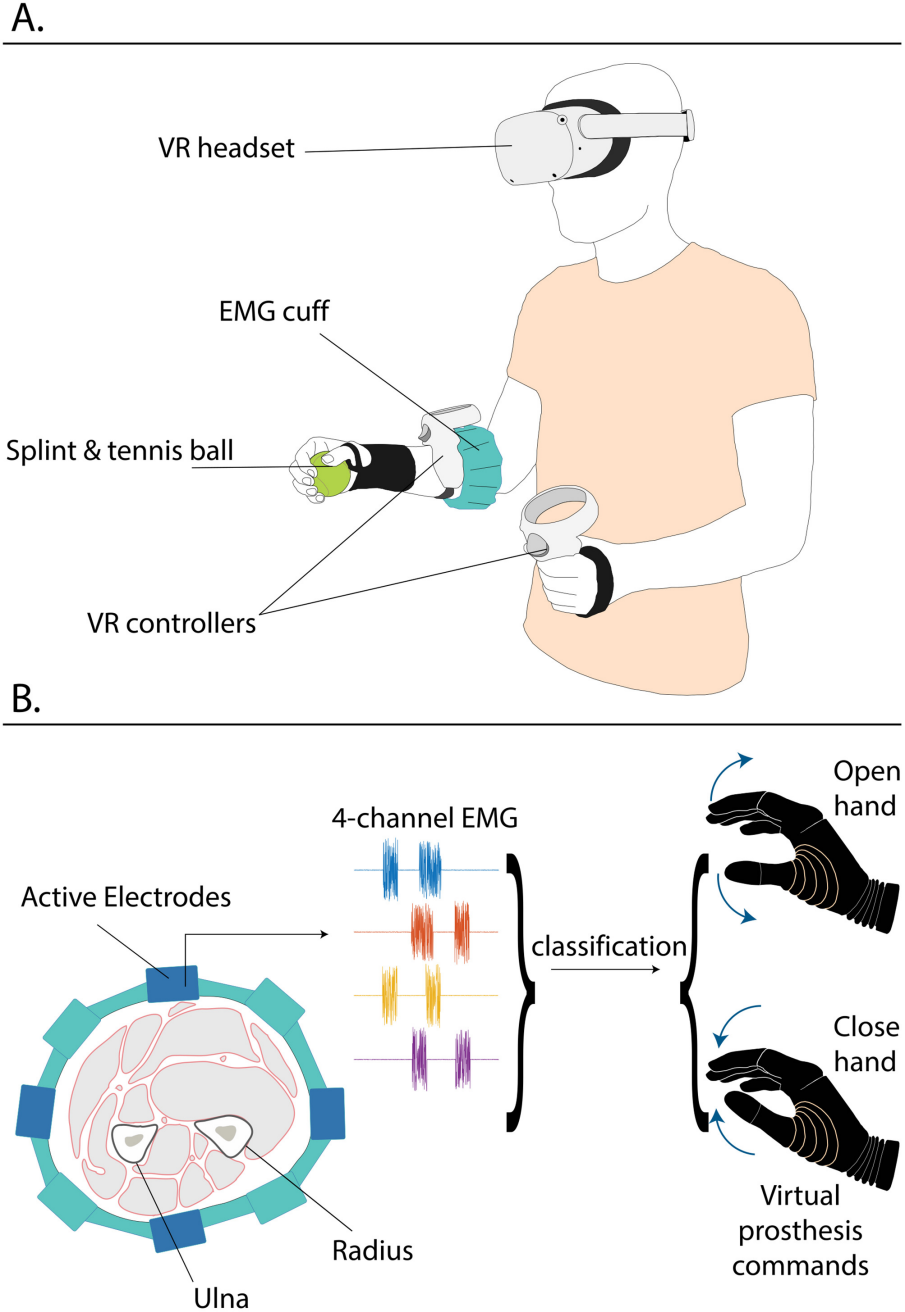
**(A)** A participant wearing the complete setup. **(B)** Schematic of the 4-channel EMG signal generation by the forearm muscles recorded by the active EMG cuff electrodes, used as an input to the classifier, which, in turn, controls the opening and closing of the virtual prosthesis in the game.

The components and their placement are displayed in Figure 1A. The elastic cuff was placed high on the participants’ forearms, approximately 10 cm distal to the elbow. A highlighted marker line on the cuff was to be aligned with the ulna, to avoid the placement of electrodes directly over the bone. The electrodes recorded the surface EMG from the forearm muscles and relayed it to the cuff controller. During the measurements, the controller was connected to the VR headset via Bluetooth.

The headset was placed onto the participants, while ensuring that the latter experienced no physical discomfort and could clearly see the VR display. The left VR controller was placed in the left hands of the participants, while the right controller was strapped around their right forearms, directly distal to the electrode cuff, as displayed in Figure 1A.

The participants wore the orthopedic splint on their right wrist to restrict wrist flexion and extension and enforce isometric contractions in the forearm muscles (27). After wearing the splint, the participants grasped the tennis ball in their right hands (Figure 1A), which were then secured with the medical tape around the ball, to emulate the presence of a grasped object, which pilot tests indicated would offer a more natural experience. Additionally, the pattern separability of EMG signals of able-bodies participants in similar experiments has been shown to more closely resemble that of people with an upper limb amputation (28). The participants stood in the middle of a designated laboratory area (approximately 3-by-3 meters), cleared of obstacles. The experimenters were seated outside of the designated area, monitoring the virtual environment on the computer, to which the VR headset was streaming its display. That way, the experimenters could see what the participants were seeing at any given time and provide necessary instructions for the completion of the experiment.

### VR game

**Figure 2:**
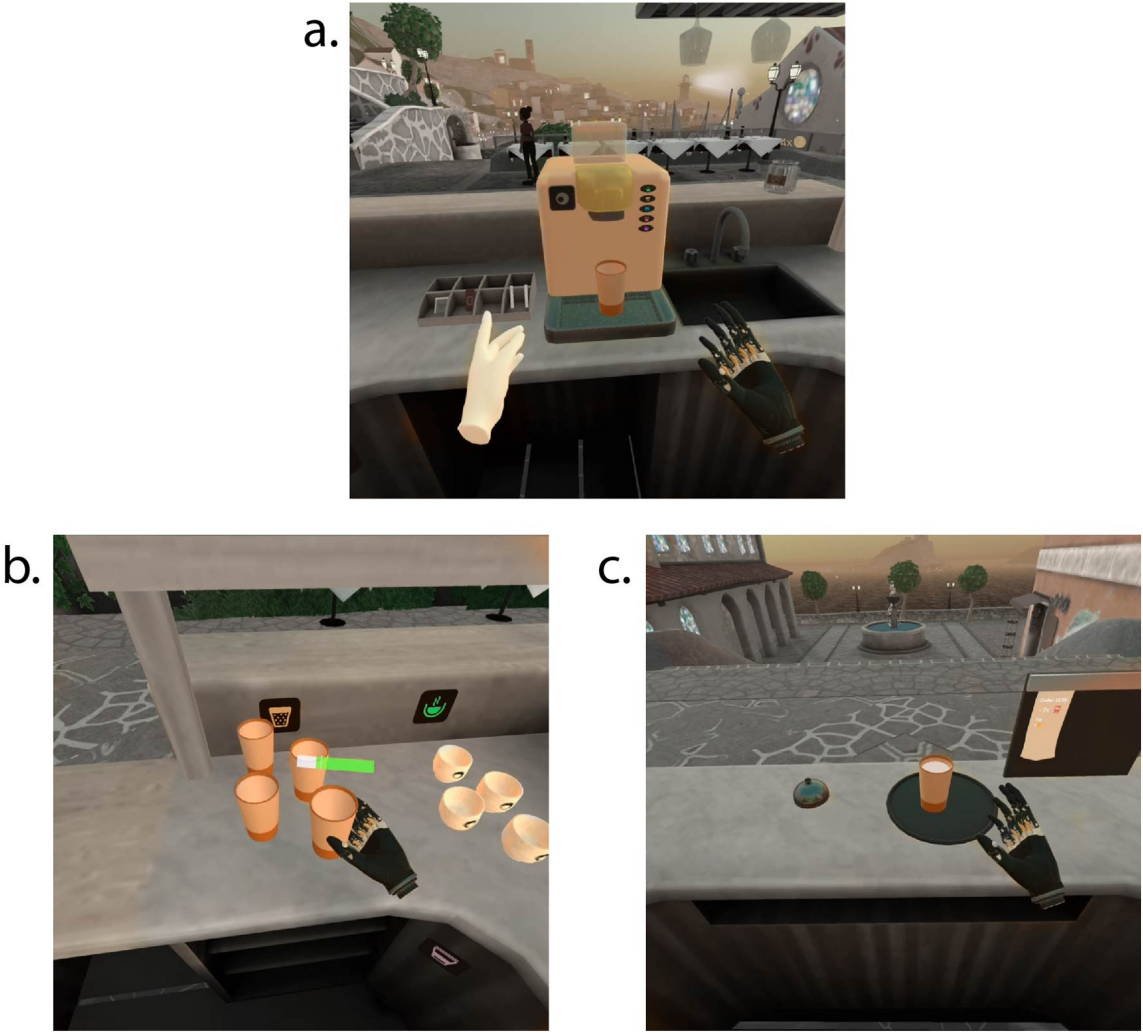
Representative screenshots from the VR game. The colors in the pictures have been partly desaturated to highlight the relevant elements. **(a)** View of the coffee machine, with a latte cup placed on it, **(b)** view of the spawning area, with latte and cappuccino cups visible, as well as the force feedback bar over the prosthesis, **(c)** view of the tray with a full latte cup, the bell, and the order slip.

The VR game placed the participants in the role of a bartender at a seaside Mediterranean café, as seen in Figure 2. Within the VR environment, the participants' avatars had a regular left hand, controlled with the triggers on the left VR hand controller, and a prosthetic right hand, controlled with pushing their right hand against the splint, by contracting their forearm muscles attempting a wrist movement. The EMG produced from these contractions was recorded by the cuff and was used to move the virtual prosthesis. Wrist flexion and extension would close and open the virtual prosthesis, respectively. The control scheme was proportional; thus, a larger muscle contraction would cause the virtual prosthesis to move faster and apply a larger force on grasped objects.

Importantly, the game has the players operating a virtual prosthesis and executing tasks with it, rather than simply training a myoelectric interface in an abstract fashion (14) as it has been shown (4) that task-specific training yields more effective skill transfer by the end of the training regimen.

**Figure 3:**
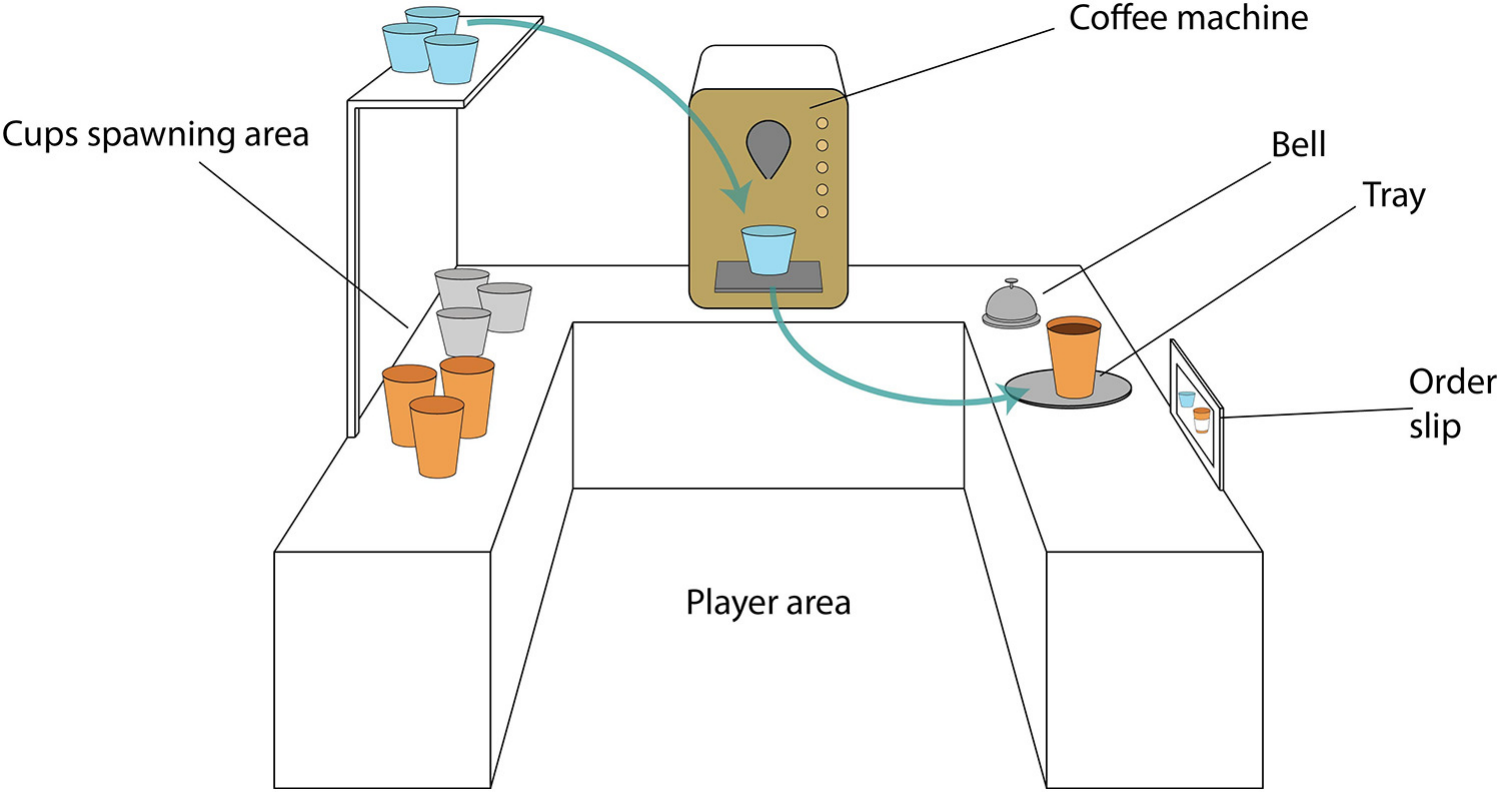
Schematic of the virtual reality game arena. The spawning area is located on the left of the coffee machine, while the tray, bell and order slip are located on the right. The participants had to transfer cups from the spawning area to the coffee machine, brew the correct beverage and transfer the filled cups to the tray. The types of beverages that had to be brewed were shown on the order slip.

Figure 3 displays a schematic of the game environment configuration. Different cups spawned on the left-side counter (see also Figure 2b), a coffee machine was located on the middle counter (see also Figure 2a), while a tray, a bell, and a vertical board could be found on the right-side counter (see also Figure 2c). There were five different types of cups, corresponding to different beverages: latte, cappuccino, espresso, “to-go” cups, and glasses. Each type corresponded to a symbol, visible over the spawning areas of each cup and on five different buttons on the coffee machine. The first three types were rigid and unbreakable. The “to-go” cups and the fragile glasses could deform or shatter, respectively, if grasped with too much force. For the needs of this experiment, the deformation of the “to-go” cups was only visual, without the requirement of a specific grasping strategy; however, the glass cups did require careful grasp force application, made possible with precise modulation of the participants' muscle contractions. If the spawning area ran out of a specific type of cup, an additional one spawned automatically.

**Figure 4:**
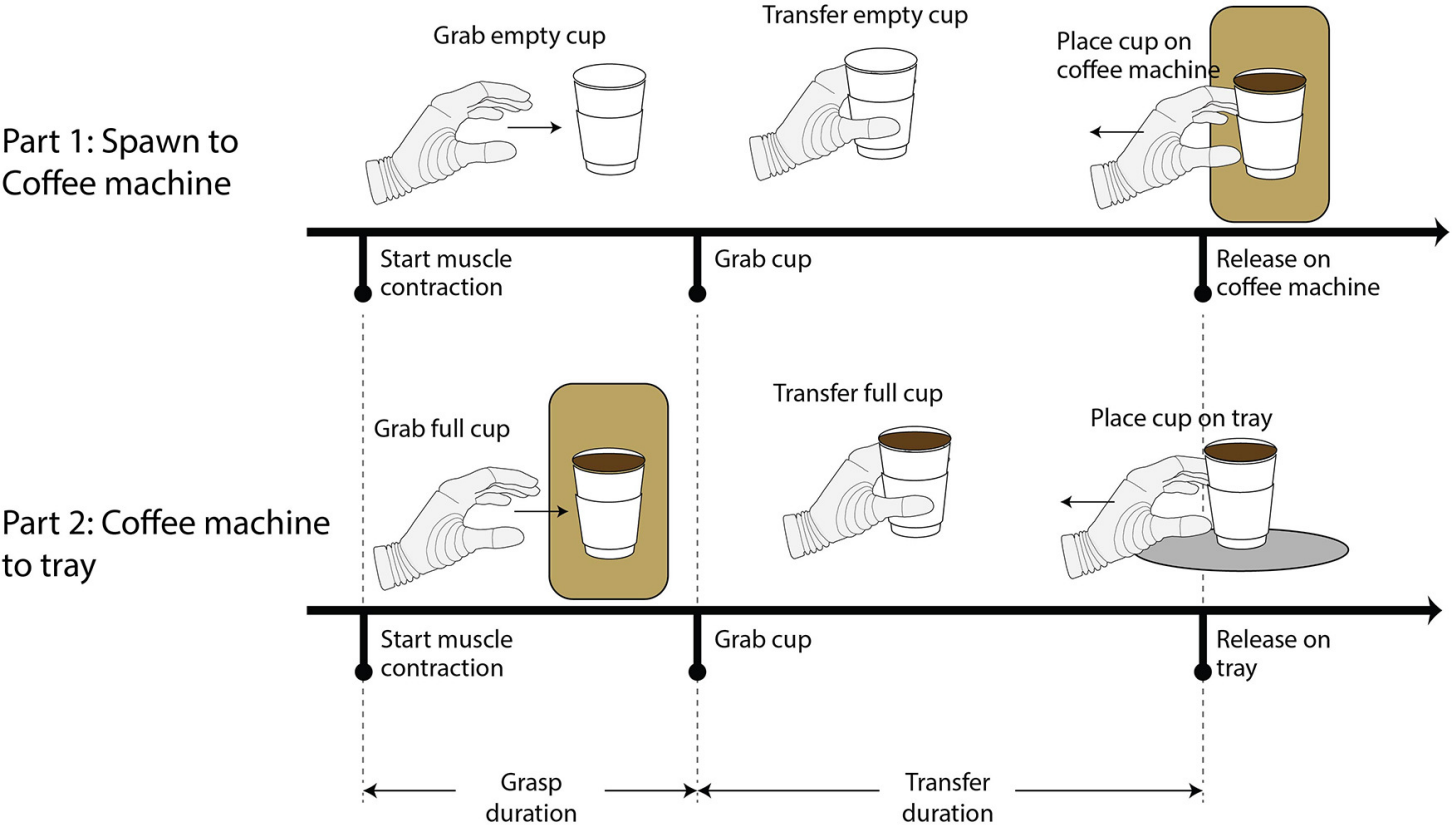
Representation of the complete preparation of a beverage. The two parts of the task are displayed, as well as the definitions of the grasp and transfer duration for each of the two parts. The cessation of muscle contractions is not shown in the figure, as it is not relevant to the calculation of the performance metrics.

During the game, the avatar of a waitress appeared behind the right-side counter and placed order slips on the board, specifying which beverages the participant had to prepare. As soon as they received the order, the participant had to use the myoelectrically-controlled virtual prosthesis to grasp the correct cup from the spawning area in the left-side counter, transport it under the nozzle of the coffee machine, use the prosthesis to press the button that would serve the correct beverage, and, once it was ready, pick up the cup again with the prosthesis and place it on the tray on the right-side counter (the steps are illustrated in Figure 4). Once all the drinks in an order were placed on the tray following this procedure, the participant rang the bell to alert the waitress, who approached the right-side counter.

If the correct beverages had been prepared, the waitress indicated this with a thumbs-up, replaced the order slip with a new one, and walked away with the now full tray. If not, the waitress indicated which beverages were missing in a text bubble above her head. Any beverages that were erroneously prepared vanished from the tray and the participant had to correctly complete the order by preparing the missing beverages.

The game had three levels of difficulty. In level one, the orders included only the first three cup types and consisted of two orders of one and three beverages, respectively. Level two increased the difficulty by introducing the last two cup types, training the participants in the modulation of the grasping force. In level three, the empty espresso cups and glasses were placed on a shelf over the left counter and the to-go cups were placed on a shelf below it, thus allowing for the evaluation of the control of the prosthesis in three different arm positions. Levels two and three consisted of three orders of two, three, and four drinks, respectively.

A force feedback scheme was implemented within the game environment, to inform the participants of the grasping force that the prosthesis applied on the various objects. Force feedback has been routinely used in literature (29–32), aiming to artificially close the prosthesis control loop, since there is no direct sensory input that corresponds to the prosthesis state. Artificial feedback loops have been shown to be beneficial in improving prosthesis control in terms of force application accuracy, speed, and robustness (27, 32, 33), while the integration of feedback in a prosthesis control interface (invasive and non-invasive) has been reported to promote feelings of embodiment in users [e.g., (34, 35)].

Force feedback in this game was implemented in the form of a color bar that appeared directly over the prosthesis whenever it approached and grasped an object (see Figure 2b). The length of the color bar was proportional to the force that the virtual prosthesis applied. Using the information provided by the force feedback, the participants could know when an object had been securely grasped with the prosthesis as well as if they needed to modulate (increase or decrease) the grasping force. Additionally, the force feedback bar made the participants aware that a cup had been securely grasped by the virtual prosthesis.

If the participant dropped a cup on the ground (e.g., by inadvertently opening the prosthesis) or crushed a glass cup by applying too much force, they had to repeat their attempt until they could successfully prepare a beverage and transport it to the tray.

### Myoelectric control

In the VR game, the participants controlled a virtual prosthesis via a myoelectric control interface. Their surface EMG was recorded by the electrodes embedded in the EMG cuff, which was placed around the proximal part of the participants' forearms (see Figure 1A), thus recording signals arising from the bellies of muscles associated with wrist and hand motion. Notably, the cuff contained 8 electrode pairs, but for reasons of simplicity, the signals of only 4 of them were used for this experiment. Therefore, when a participant was wearing the electrode cuff, the active electrodes were positioned around their forearm in a cross-shape (see Figure 1B).

The 4-channel EMG signal was routed to a controller, which was responsible for processing the EMG signals generated by the participants. Using an LDA classifier, the controller translated myoelectric signals to prosthesis commands (Figure 1B). These commands were scalable, i.e., a smaller muscle contraction would result in the prosthesis moving slower and vice versa, thus implementing proportional control of the virtual prosthetic hand. During the experiment, the classifier output was transmitted wirelessly over Bluetooth to the VR headset at a frequency of 10 Hz, where it was used by the game software to drive the virtual prosthesis in the training game.

The classifier had to be trained and calibrated at the beginning of each experimental session, to accommodate each new configuration. The calibration was performed with the use of “MyoPlus”, a dedicated mobile app, developed by OttoBock. These signals were used to train the LDA classifier (a procedure that lasted for a few seconds after the signal recording had been completed) and generate the muscle activation patterns later used for controlling the prosthesis.

### Experimental procedure

#### General structure

The participants had to perform five experimental training sessions on five consecutive days to complete the experiment. As will be detailed below, these sessions included the calibration of the EMG cuff and the VR headset and the training itself. As far as it was possible, the duration of the sessions was kept below 1 h, with the training itself lasting at most 30 min. During the training, the participants had to play the VR serious game presented above in three levels of difficulty (provided they did not surpass the 30-min limit), under the supervision of the experimenters.

It should be noted that the measurements presented in this paper were executed as part of a larger experiment regarding skill transfer from the VR game to the use of a real myoelectrically-controlled prosthesis following the training. The results of that study are beyond the scope of this work and are presented in a separate publication. For the needs of the overall study, besides the tasks presented here, the participants performed a pre-test before their VR training in the first session and a post-test after the VR training in the last session. In these tests, the participants used a prosthesis simulator to perform a modified Box and Blocks test (32), three tasks of the SHAP test (36), a cylinder test (4). Additionally, they were questioned on their workload and motivation in their second and fifth sessions. However, the current work will focus on the VR training that the participants received in between the testing sessions.

#### First session

The participants were given the information letter concerning the experiment to study ahead of time. The experimenters went through the information again with each participant on the day of their first training session. Once the participants had fully understood the procedure and goal of the experiment, they signed an informed consent form and filled in the MSSQ, to ascertain their eligibility for participation. The rest of the first session and the rest of the four sessions of VR training followed the same structure comprising a calibration and training step, as illustrated below.

#### EMG calibration

The myoelectric cuff was placed on the right forearm of the participants, followed by the wrist splint. The tennis ball was placed in the palm and secured with medical tape, immobilizing the fingers around the tennis ball. Using the MyoPlus interface on the tablet, the classifier was calibrated to the new configuration of the myoelectric cuff. The participants were required to stand and follow a set of instructions shown on the interface. The participants assumed three different arm positions (elbow bent at 90 degrees and forearm parallel to the ground, arm hanging vertically next to the torso, and arm extended forward). In each position, they were asked to relax, flex, and extend their wrist against its constraints, while their EMG signals were recorded by the controller. These signals were used to train the LDA classifier (a procedure that lasted for a few seconds after the signal recording had been completed) and generate the muscle activation patterns later used for controlling the prosthesis. The quality of the calibration was evaluated empirically, based on the overlap of the generated patterns on a spider plot displayed in the MyoPlus interface. The procedure was repeated, potentially after shifting the electrodes around the participants' forearm, if the patterns were deemed too similar, which would hamper the recognition ability of the classifier.

#### Preparation of VR setup

Once the myoelectric interface was calibrated, the participant was moved to the center of the dedicated play area. The left VR controller was placed in their left hand and the right controller was strapped onto their right forearm directly distal to the EMG cuff, using an elastic Velcro band. The right controller was used by the game to project the position of the virtual prosthesis to that of the participant's real hand. After activating the VR headset, the experimenters placed it on the head of the participant.

The participant was then instructed to calibrate the VR headset by setting the floor level and drawing the guardian to correspond to the dimensions of the play area. After this initial calibration, they were instructed to launch the VR prosthesis training game.

#### VR environment calibration and gameplay

The participant calibrated the VR game, guided by the experimenters and the instructions inside the VR environment as follows: using the left controller, they indicated that the virtual prosthesis was to be on the right side and the control input would come from the EMG cuff. Then, they assumed a stance with their arms outstretched in front of them, so that the system could calibrate the position of the virtual prosthesis, which was to be projected ahead of the right VR controller (placed on the forearm), to a position that had to be consistent and overlap with that of the participant's real hand. Once the calibration was complete, the participant was asked to look at the virtual prosthesis and report if its position was indeed the perceived position of their real hand. If necessary, this procedure was repeated, until this overlap was achieved.

The participant then followed the instructions to connect the EMG cuff to the VR headset via Bluetooth. Once connected, the participant was instructed to test the connection by flexing and extending their wrist against the splint. Movement of the virtual prosthesis in response to these muscle activations indicated that the myoelectric control interface had been established and the training could begin. The participants were then presented with the main menu of the training game, where they could select which difficulty level of the game they wished to play. In the first two sessions, the participants started with level 1, followed by levels 2 and 3, while in subsequent sessions they started with level 2, followed by 3 and, finally, level 1. The maximum duration of the training sessions was 30 min (from the onset of the first level played). A level was not launched if the participants had already surpassed the 30-min limit.

### Data analysis

To clarify the terminology that will be used, it is important to note that for the analyses the experimental task was implicitly divided into two parts, as shown in Figure 4: the first part includes grasping a cup from the spawning area and releasing onto the coffee machine and the second part includes grasping from the coffee machine and releasing onto the tray. The following outcome measures were considered:

• Grasp duration: as illustrated in Figure 4, it is defined as the time between the first activation of the prosthesis until the moment the cup is grasped (for grasping at the spawning area and at the coffee machine). It is defined for each of the two parts of the task independently. The myoelectric control scheme and the related skill development can be evaluated based on this measure. The risk of spilling the beverage when grasping from the coffee machine was thought to affect the execution of the task, which is why its two parts were evaluated separately.
• Transfer duration: as illustrated in Figure 4, it is defined as the time between the grasp of the cup and its release onto one of the target areas (the coffee machine or the tray). It is defined for each of the two parts of the task independently, as a cup full of liquid was thought to affect the way that the participants would manipulate it. This measure can provide insight into how the participants adapted to the altered physics of the virtual world, as they received the same proprioceptive feedback in both parts of the task even though the cups were full when picked up from the coffee machine. Notably, the distance over which the participants had to transfer the cups was comparable in the two phases of the task; therefore, the same duration was expected for transfer duration.
• Glass cup break rate: the percentage of glass cups crushed with the prosthesis, over the total number of attempts to grasp a glass cup. Force feedback was designed to facilitate the modulation of the grasping force; therefore, this measure can highlight the efficacy of the feedback and the degree at which the participants managed to utilize it.
• Grasp submovements for glass cups: the number of prosthesis activations prior to successfully grasping a glass cup. A smaller number of submovements would indicate a fluid and more controlled movement and, therefore, a higher amount of acquired skill. The different arm positions that were required for picking up the cups from the spawning area may have affected the execution of the task, which is why the measure was considered separately for the two parts of the task.

The outcome measures mentioned above were considered for each of the participants in their first and fifth training sessions. If a value on either session was missing, the one corresponding to the day immediately after or before, respectively, was considered.

Outlier values beyond ±1.5 times the interquartile range were removed. The Shapiro–Wilk test was used to test the datasets for normality, several of which were found not to follow a normal distribution. For this reason, nonparametric statistical tests were used for further analysis, namely the Friedman and Wilcoxon signed-rank tests, to assess the effect of the training as well as the influence of the virtual environment on the performance. The Friedman test was performed on the grasp and transfer duration outcome measures, wherein within-subject factors were the cup type (latte, cappuccino, espresso, to-go, and glass), the session (beginning or end of the training), and the task phase (whether transferring from the spawn to the coffee machine or from the coffee machine to the tray). The Bonferroni correction was used to correct for the multiple comparisons when evaluating the differences among cup types. The Wilcoxon test was used to evaluate the main effects of the grasp and transfer durations *post-hoc*, as well as the glass crush rate and number of submovements outcome measures.

Finally, a larger number of grasp submovements is indicative of lack of precise control of the myoelectric signal that the participants generated. We hypothesize that this kind of erratic myoelectric control would also result in more frequent crushes when attempting to grasp glass cups. Therefore, in order to establish this relation, we obtained the Kendall correlation coefficient between the crush rate and the number of submovements.

Notably, the deformation of the “to-go” cups was not considered in the analysis, as it solely affected the visual aspect of the game, with no functional differences in grasping and transferring them compared to the rest of the rigid unbreakable cups. The level of statistical significance for all tests was set at *α* = 0.05.

## Results

### Grasp duration

**Figure 5:**
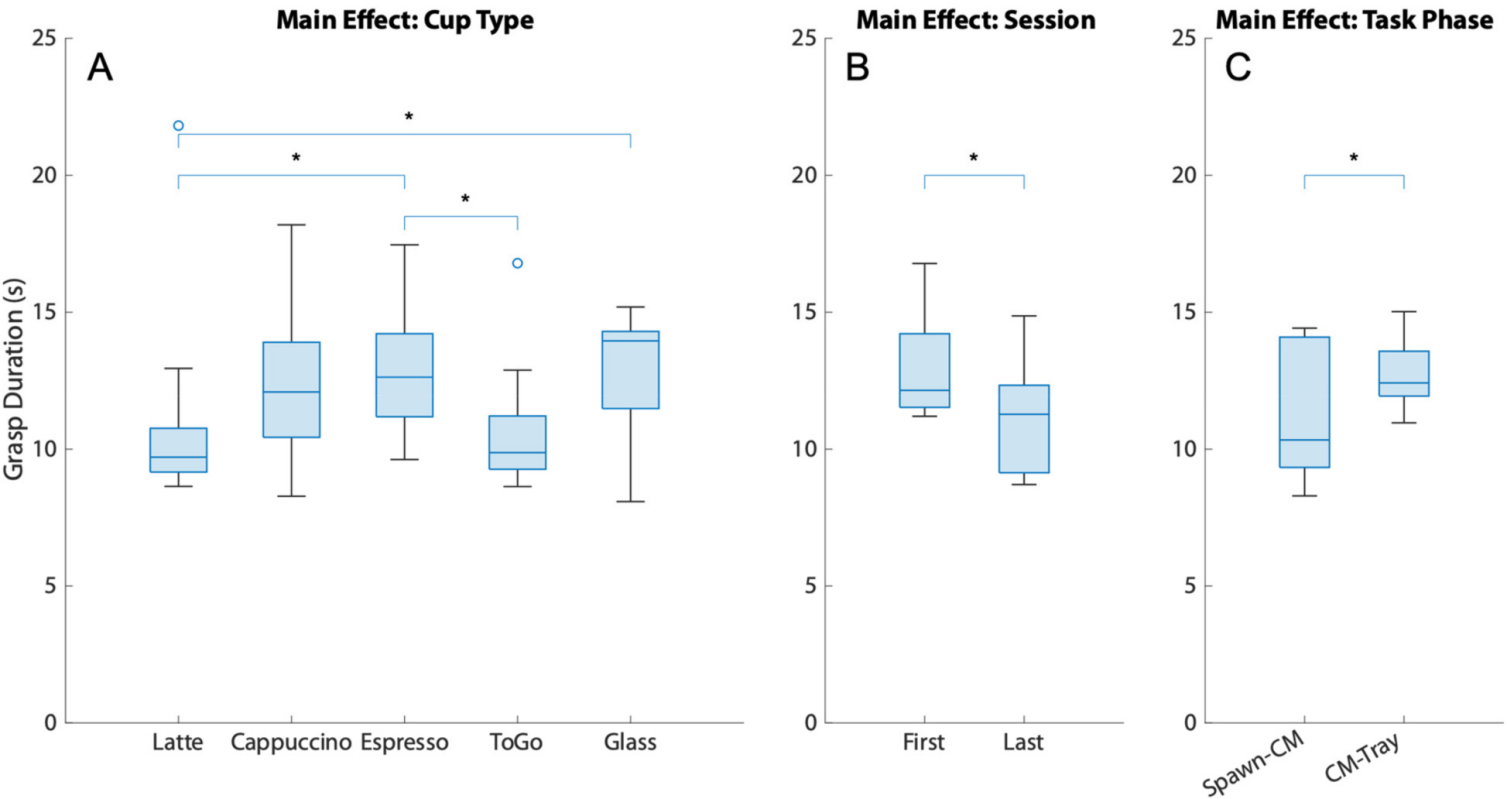
The main effects of the grasp duration. **(A)** Cup Type, **(B)** Session, and **(C)** Task Phase. Lines and asterisks denote significantly different pairs. (CM stands for Coffee Machine).

Figure 5 displays the main effects of the transfer duration (A. Cup type, B. Session, and C. Task phase). The Friedman test showed a significant effect of the cup type on the grasp duration (*χ*^2^ = 9.8, *df* = 4, *W* = 0.306, *p* = 0.044); however, no significant differences between pairwise comparisons of constituent cup types were found *post-hoc* after the Bonferroni correction. The training session and the task phase both had a significant effect on the grasp duration. Specifically, it was significantly reduced in the last training session (Figure 5B, *z* = 2.191, *p* = 0.027, *r_B_* = 0.782) compared to the first training session. It was significantly higher when grasping the cups from the coffee machine to transfer to the tray (Figure 5C, *z* = −2.09, *p* = 0.037, *r_B_* = −0.745) compared to the phase of moving the cups from the spawn area to the coffee machine.

### Transfer duration

**Figure 6:**
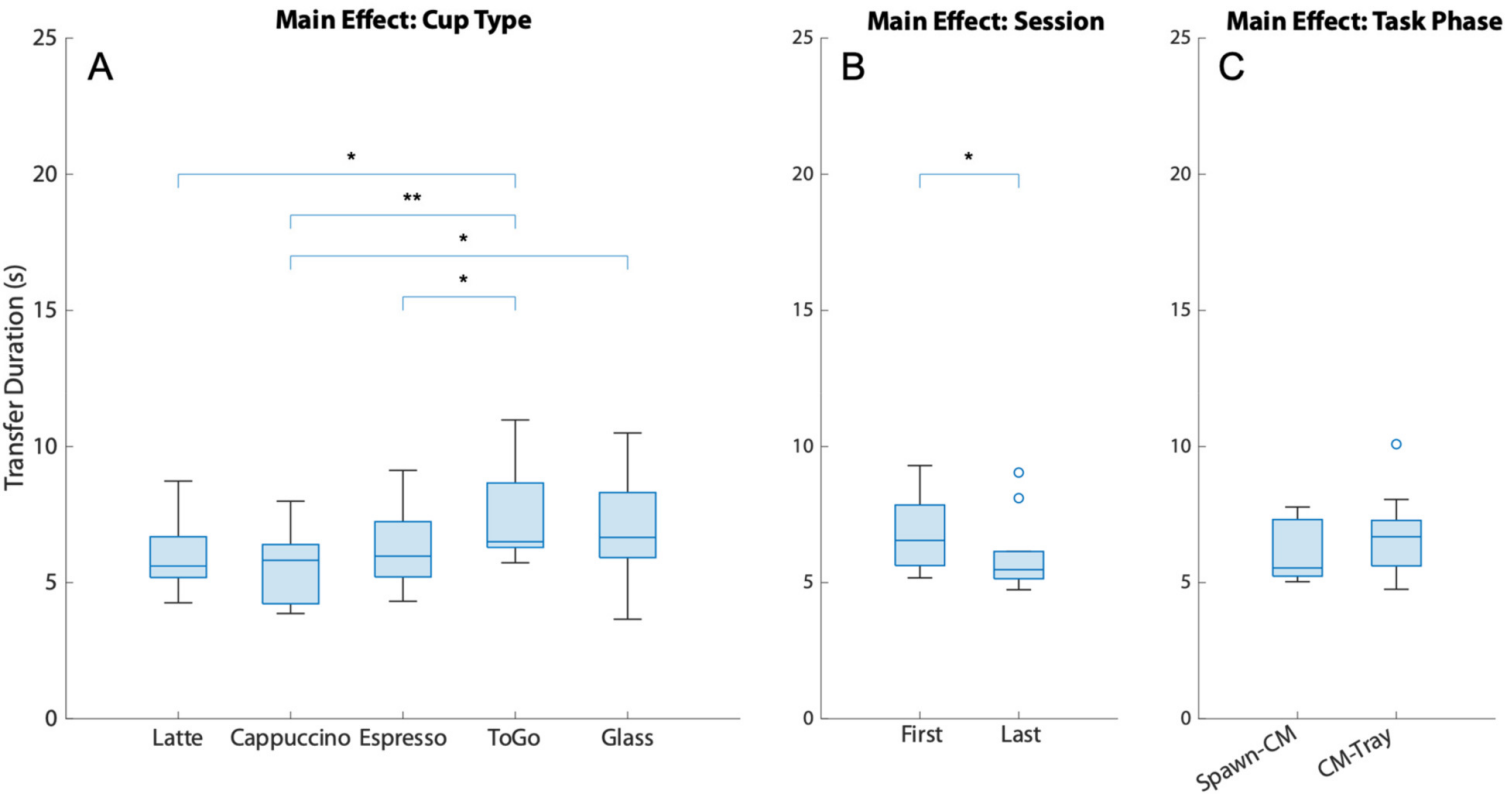
The main effects of the transfer duration. **(A)** Cup Type, **(B)** Session, and **(C)** Task Phase. Lines and asterisks denote significantly different pairs. (CM stands for Coffee Machine).

Figure 6 displays the main effects of the transfer duration (A. Cup type, B. Session, and C. Task phase). The analysis showed that the type of cup the participants grasped had a significant effect on the transfer time (*χ*^2^ = 14.96, *df* = 4, *W* = 0.374, *p* = 0.005), with the only cup pair to display a significant difference in transfer time after the Bonferroni correction was that of the cappuccino and ToGo cups, as shown in Figure 6A (*z* = −2.803, *p* = 0.02, *r_B_* = −1). The transfer time appeared to have significantly decreased by end of the training (Figure 6B, *W* = 35, *z* = 3.38, *p* = 0.016, *r_B_* = 0.944), while the task phase was not significantly affected (Figure 6C, *W* = 13, *z* = −1.125, *p* = 0.301, *r_B_* = −0.422).

### Glass cup breaks and submovements

**Figure 7:**
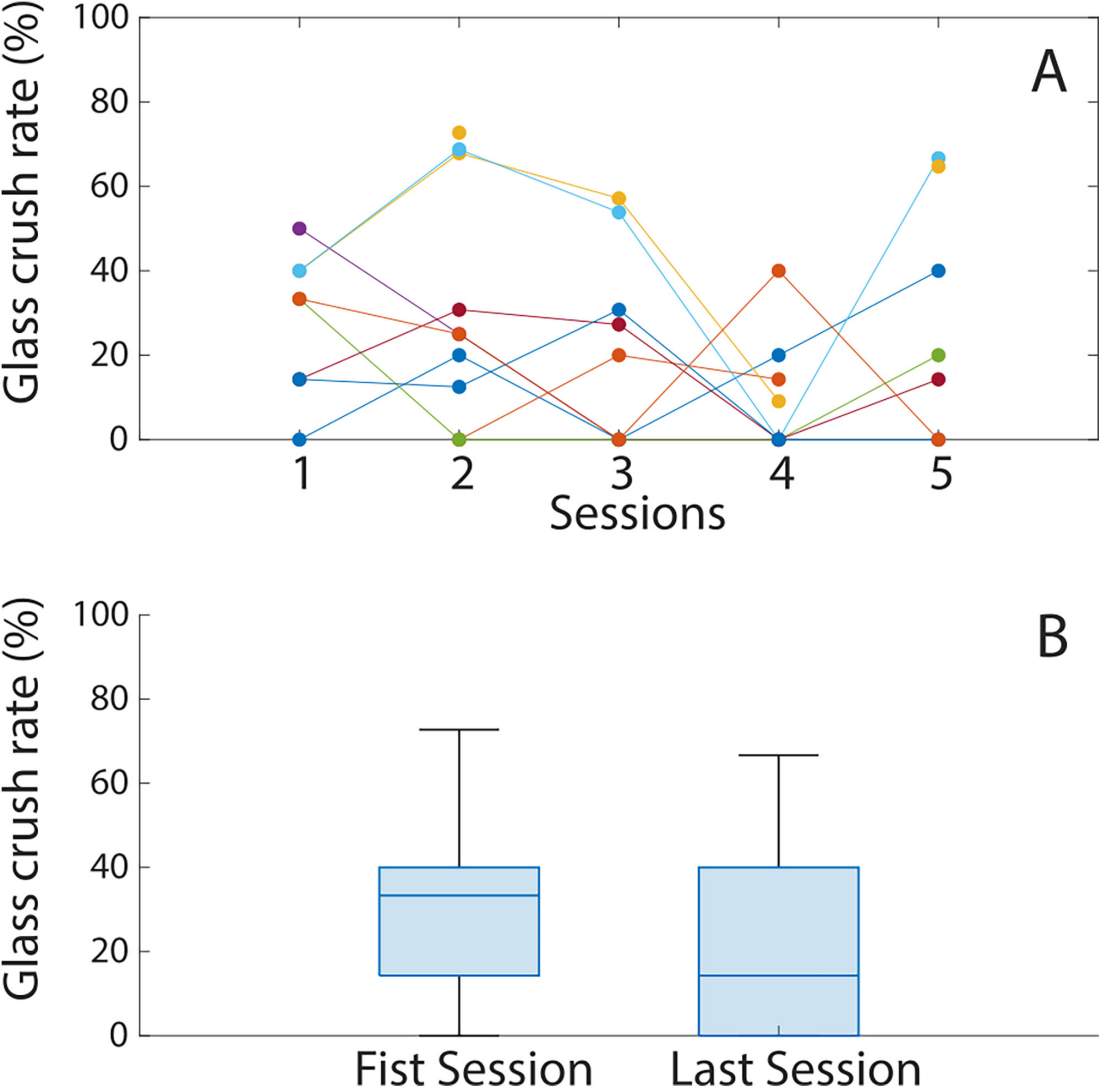
**(A)** The glass cup crush rate of each participant over the five training sessions. Each colored line corresponds to a different participant. **(B)** The glass cup crush rate distribution in the first and last training sessions.

Figure 7 shows the percentage of attempts where a glass cup was crushed over the total number of attempts at grasping a glass cup for individual participants. There is no significant change (*z* = 0.711, *p* = 0.514, *r_B_* = 0.267) in the rate at which the participants crushed the glass cups, which indicates that despite the training, the precision of the prosthesis force application and modulation did not change for this type of cup.

**Figure 8:**
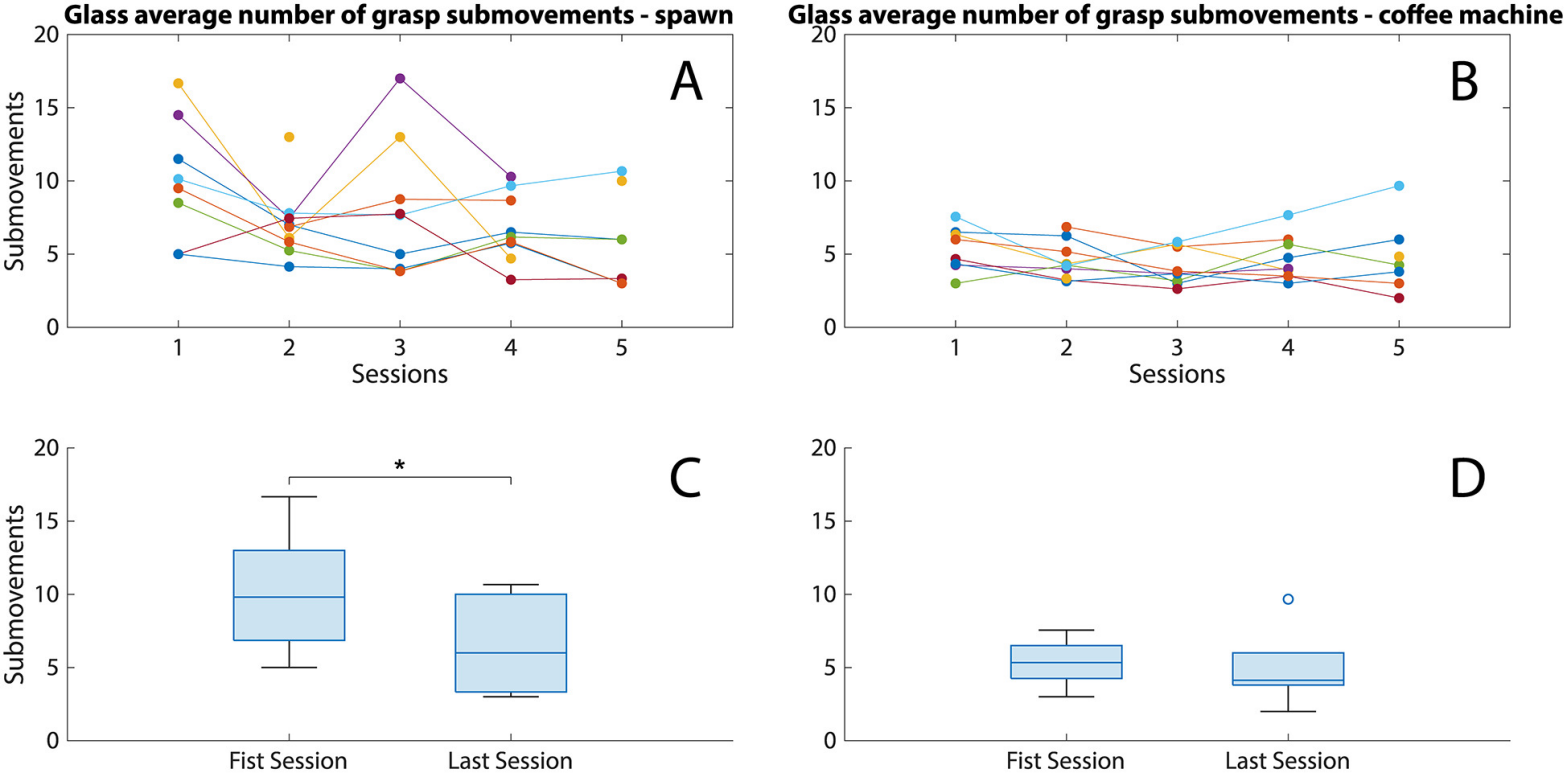
**(A,B)** The number of submovements when grasping a glass cup from the spawning area **(A)** and the coffee machine **(B)** in the five training sessions. Each colored line corresponds to a different participant. **(C,D)** Boxplots presenting the number of submovements distribution in the first and last sessions for grasping from the spawning area **(C)** and the coffee machine **(D)** Statistically significant pairs are denoted by asterisks (*p < 0.05).

The number of prosthesis activations when grasping a glass cup from the spawning area is significantly reduced (*z* = 2.395, *p* = 0.014, *r_B_* = 0.855) by the end of the training (Figure 8C). In Figure 8A it can be observed that the number of submovements when grasping a glass cup from the spawning area appears to plateau as early as in the second session. The same metric showed no change when grasping glass cups from the coffee machine (Figure 8D, *z* = 0.968, *p* = 0.375, *r_B_* = 0.345); however, the number of activations was already low in the first session with its median being comparable to that of grabbing from the spawning area in the last session. Lastly, it appeared that the number of submovements in all sessions (8B) already lay within the plateau seen in Figure 8A.

The Kendall's tau between the crush rate and the submovements in both parts of the task in the first and last training sessions can be seen in Table 1. There is largely low to no correlation between these metrics, apart from the crush rate and the submovements during grasping from the spawn area which are only weakly correlated in the last training session (*τ* = 0.524).

**Table 1:** Kendall correlation coefficients between the crush rate and the number of submovements. The blue and white boxes denote Kendall's tau and its corresponding *p*-values, respectively.

	First training session			Last training session		
	Crush rate	Submovements— spawn	Submovements— coffee machine	Crush rate	Submovements— spawn	Submovements— coffee machine
Crush rate	1	*τ* = 0.094	*τ* = −0.303	1	***τ* = 0.524**	*τ* = 0.448
Submovements—spawn	*p* = 0.055	1	*τ* = 0.045	***p* = 0.043**	1	*τ* = 0.493
Submovements—coffee machine	*p* = 0.237	*p* = 0.857	1	*p* = 0.107	*p* = 0.72	1

A bold font denotes significant values.

## Discussion

This study explored the effect that different elements of a virtual reality environment have on user performance in a prosthesis control training rehabilitation serious game. This knowledge could provide insight into ways the VRE can be exploited to optimize prosthesis control training and make the training scheme implemented through the game more engaging. The training system was evaluated based on the performance of able-bodied participants as it evolved over five sessions, carried out over consecutive days. By examining the outcome measures, it is possible to appraise the efficiency of the specific training parameters that were used, namely the characteristics of the game that the participants played and, by extension, identifying ways that the virtual reality framework can potentially be exploited in ways that better benefit the rehabilitation and training outcomes.

The use of serious games has been investigated extensively in scientific literature as a means for motor rehabilitation and, more specifically, of prosthesis control training. Despite a consensus regarding their standardization being still under investigation (37), the engaging and entertaining nature of game-based training protocols have been shown to improve user performance over the duration of a training regimen (4, 28, 37, 38). Moreover, and most importantly, this approach to rehabilitation has been shown to be accompanied by skill transfer to real-world prosthesis use, such as in the case of (39), in which a pattern recognition-based control scheme was utilized, similar to the one used in this study. As such, it was expected that there would be an observable improvement in the performance of the participants by the end of the training.

Additionally, a system that integrates an engaging VR serious game with a simple and easy-to-use myoelectric interface can be used at home, with the presence of a therapist not being necessary and at the leisure of the patient. These considerations informed the design of the system that was used in this study, with the myoelectric interface being simple to don and doff as well as straightforward to train. Arguably, a four-electrode LDA interface may seem excessive; however, its use serves two purposes. First, for a non-professional, it is easier to set up than a purely proportional interface, which would require careful and precise placement and configuration of surface EMG electrodes. In the current configuration, the use of pattern recognition renders the system more user-friendly. Second, a future version of a similar system can make use of the extra electrodes to implement a more complex myoelectric control interface, of multiple DoFs, thus future-proofing the configuration. The provision of force feedback within the VR environment in lieu of a more conventional delivery pathway such as through vibrotactile stimulation also adds to the simplicity of the configuration. Specifically, regarding the completion time of the constituent elements of the coffee making task, in this case exemplified by the grasp and transfer durations, the participants could transfer the cups significantly faster at the end of their training. This indicates that the latter had an observable and significant effect on the way the participants manipulated the virtual objects. Conversely, whether the cups were full or not (as exemplified by the task phase) did not seem to have an effect. The type of cups, however, had a significant omnibus effect on the transfer duration, although, as mentioned, the exact mechanism of this effect could not be isolated *post-hoc* Despite not identifying the effects of individual cup types, the results suggest that in a game of this type a user would have to adapt to accommodate the properties of the virtual objects, with the game, thus, offering training in different control schemes, enhancing the rehabilitation regimen. The training also had a beneficial effect on the grasping of the virtual cups, with the grasp duration being significantly shorter at the end of the training, indicating the development of a new motor skill as a result of the training. Additionally, the participants appeared to take significantly longer to grasp the cups from the coffee machine, when they were full. This could, again, indicate that the physics of the virtual world, in this case manifested in the mechanics of the liquid in the cups, affected the way that the participants controlled the virtual prosthesis, as they performed a grasp much more slowly and in a more controlled manner, to not spill the beverages (and, thus, have to repeat the preparation of the beverage from the beginning). As for the game tasks (as they were illustrated in the Introduction as one of the four aspects of the game design evaluated in this study, and, in this case, the different cup types), they also seemed to have an overall effect on the grasp duration, indicating that the performance of the participants was affected by the difficulty levels offered by the game. Therefore, it can be argued that the design capabilities of VR can be exploited for the development of tasks of increasing difficulty, each targeting a specific motor skill.

Despite the training, the participants did not manage to reduce the rate at which they crushed the glass cups. In other words, the participants were inconsistent in their ability to precisely modulate the grasping force of the virtual prosthesis. To achieve that, they would have to rely on incidental sensory information, such as the sound of the prosthesis and their own muscle proprioception as well as the force feedback delivered to them during gameplay, which was a direct and objective representation of the force applied on grasped objects. This result may be an indication that the force feedback provided to the participants was not effective, as it did not offer an improvement in their ability to accurately modulate and control their force. A feedback scheme that communicates the same grasp force information to the participants in a more direct or intuitive way such as the ones presented in (40) might have been a better solution in this case. Of course, the use of the VRE can offer more varied opportunities in the way the feedback is delivered, e.g., a more obvious force bar or the change of the object colors in response to the grasp force.

It is important to keep in mind that the ultimate goal of any serious game is to bring about quantifiable transfer of prosthesis control skills from the VR environment to the operation of a real, physical myoelectric hand prosthesis. It can be argued that the ineffectiveness of the force feedback (even within the VR environment) could not have been instrumental in the development, much less the transfer of a novel motor skill from the VRE into the real world. Nevertheless, this training has been shown to achieve skill transfer comparable to a similar training regimen using a physical prosthesis; however, skill transfer is beyond the scope of this work.

The participants grasped glass cups from the spawning area with a smaller number of prosthesis submovements by the end of the training, showcasing that they could eventually operate the prosthesis in a more controlled and fluid manner. This behavior was achieved and plateaued as early as in the second training session. As mentioned, the submovements when grasping full cups from the coffee machine (second part of the task) appeared to already lie within this plateau (Figures 8A,C), with little to no change. For this reason, one could argue about the benefit of this part of the task to the overall training. Possibly, the focus of the game should be shifted towards tasks that train an aspect of control that requires the learning of a new and possibly unintuitive motor skill.

Surprisingly, there was no correlation between the glass crush rate and the number of submovements in most of the cases, from which it can be inferred that inconsistencies in the myoelectric signal in general did not affect the virtual prosthesis force modulation. Viewed in a positive light, we can surmise that the participants could control the force applied on the glass cups despite the large number of submovements, this being an indication of the system's robustness.

Notably, the participants were not given specific instructions during the training regarding the speed at which they performed the task or a specific goal regarding their precision (exemplified by the crushes and submovements). From these results, it can be surmised that learning using this game is an emergent process, while it is possible that a more specific instruction could have pushed the participants towards a different direction, where they would focus on developing specific skills related to the task. With careful and focused guidance, the participants may be motivated to aim for specific alterations in their control of the virtual prosthesis, to improve distinct aspects of their performance.

Therefore, depending on the goals set for a rehabilitation protocol, a user can be given explicit instructions pertaining to their gameplay that implicitly target a specific motor skill. For instance, exploiting feedback to have the user focus attention on specific aspects of the task (4), may eventually impact their myoelectric control skills more directly than the unstructured training approach taken in this study. An example of such an approach would be to exploit the VRE in such a way to have the users focus on the force feedback to specifically train their precise force modulation skills.

Nevertheless, a consequence of the lack of instruction was that the participants were free to explore and navigate the virtual environment, which gave rise to the evaluation of their reaction to the way that the virtual environment functioned. The mismatch between the visual input from the VR headset and the participants' natural proprioception could be off-putting for some users, thus hindering the goal of the game (26). Notably they did not comment on any effect this mismatch had on them during their experimental sessions. This primarily speaks to the level of immersion of this particular game. Furthermore, it suggests that since changes in the virtual world physics can be accommodated by the users, the former could also be tailored to assist in the acquisition of a specific motor skill. Combined with freedom in task design, the demonstrated efficacy of this training scheme as well as the motivation on the side of the participants (22), this places virtual reality at a significant advantage compared to conventional rehabilitation methods, as training protocols can be designed to exact specifications and yield the desired rehabilitation outcomes.

This study was not without its limitations. A major limitation was the unavailability of EMG signal storage for analysis. The development of motor skills was evaluated solely based on the qualitative performance metrics mentioned above. Suitable analysis of the muscle activity would provide with more complete information regarding the control of the virtual prosthesis and the effect that the training protocol had on it. Additionally, a lack in improvement in the glass cup crush rate may be attributed to the relatively small number of glass cups that the participants had to handle overall. Thus, since the focus of the training was not on this type of cup, perhaps this training protocol was not sufficient for the participants to efficiently develop the skills necessary for precise force control and modulation.

Moreover, the non-inclusion of participants with forearm amputations is another major limitation, which, of course, will be addressed in subsequent works regarding this system, as its efficacy on the target user population, namely, people with upper limb amputations, is still to be established. Nevertheless, we do not believe that our participants being young, able-bodied and in good general health, biased the results of the study, as part of the user pool for this system does indeed fit this demographic, while myoelectric control and serious game studies have been conducted with geriatric patients with positive results (41). Of course, a similar study with participants with upper limb differences would establish if the training has similar effects in its target population with regard to motor skill development. Its usability and user motivation associated with it have already been displayed in (23).

Finally, since the game was still in development at the time of the experiments, there were several cases wherein unresolved bugs and glitches in the game's code affected the gameplay and hindered the training protocol. We did our best to work around these issues and mitigate their influence on our results. Irrespective of these issues, the results show a clear benefit of the VR training; therefore, it can be surmised that a rectification of the game's programming would greatly improve the training experience and by extension, hypothetically, even the rehabilitation outcomes.

Although not all hypotheses pertaining to the performance were verified, the results of this study indicated that certain aspects of the participants' performance did indeed improve, while our companion study (22) indicated that there was an observable skill transfer from the VR tasks to real-world ones. Therefore, based on the results of the present study, we can draw some insight regarding the design decisions in similar rehabilitation regimens, for an overview see Table 2. We have shown that the tasks executed in this VR game is a viable tool for the training of the control of a 1 DoF prosthesis. The participants were shown to adapt to the game's adapted physics, the latter of which can be exploited to guide the users to adapt their task execution strategies. A more effective feedback interface should be designed to facilitate the development of more accurate grasping force modulation by exploiting the design freedom that VR offers, while the myoelectric control scheme was simple to set up and use, making it ideal for at-home applications.

**Table 2:** Summary of the qualitative results of the study and recommendations for future game design.

Game feature	Outcome	Recommendation
In-game physics	Participants adapted to altered physics	Exploit this adaptability to guide user strategies and performance
Game tasks	Task objective and difficulty level affected performance	Implement a variety of tasks to target specific rehabilitation goals
Myoelectric control	Effective and simple 1-DoF scheme, easy to set up	User-friendly myoelectric control interfaces, ideal for at-home training
Feedback	Feedback scheme did not improve performance	Design of a smarter, more intuitive feedback scheme

The outcomes of this study give clear indications of how the game design elements affect the training and user performance. For instance, participants adapted to the altered physics of the virtual world, despite the latter being inconsistent with their proprioception. Conversely, the force feedback scheme that was used did not improve the precision of force modulation (leading to a high number of glass cup crushes), indicating room for improvement. Therefore, this paper presents the first steps in identifying how prosthesis training through a VR serious game is affected by the game design choices. The insights provided in this paper can be used as a valuable steppingstone in the design of similar games, which, combined with testing in the target population and rigorous validation, can be introduced in the clinical rehabilitation process.

## Data Availability

The raw data supporting the conclusions of this article will be made available by the authors, without undue reservation.
